# Chitosan Films Obtained from *Brachystola magna* (Girard) and Its Evaluation on Quality Attributes in Sausages during Storage

**DOI:** 10.3390/molecules26061782

**Published:** 2021-03-22

**Authors:** Juan Manuel Tirado-Gallegos, Paul Baruk Zamudio-Flores, Miguel Espino-Díaz, René Salgado-Delgado, Gilber Vela-Gutiérrez, Francisco Hernández-Centeno, Haydee Yajaira López-De la Peña, María Hernández-González, J Rodolfo Rendón-Villalobos, Adalberto Ortega-Ortega

**Affiliations:** 1 Tecnología de Productos de Origen Animal, Facultad de Zootecnia y Ecología, Universidad Autónoma de Chihuahua, Periférico Francisco R. Almada km 1, Chihuahua, Chihuahua C.P. 31453, Mexico; 2 Fisiología y Tecnología de Alimentos de la Zona Templada, Centro de Investigación en Alimentación y Desarrollo, A.C.-Unidad Cuauhtémoc, Avenida Río Conchos s/n, Parque Industrial, Apartado Postal 781, Ciudad Cuauhtémoc, Chihuahua C.P. 31570, Mexico; aster3000@hotmail.com; 3 Tecnológico Nacional de México/Instituto Tecnológico de Zacatepec, Posgrado-Departamento de Ingeniería Química y Bioquímica, Calzada Tecnológico 27, Zacatepec, Morelos C.P. 62780, Mexico; renesalgado@hotmail.com; 4 Laboratorio de Investigación y Desarrollo de Productos Funcionales, Facultad de Ciencias de la Nutrición y Alimentos, Universidad de Ciencias y Artes de Chiapas, Libramiento Norte Poniente 1150, Col. Lajas Maciel, Tuxtla Gutiérrez, Chiapas C.P. 29000, Mexico; gilber.vela@unicach.mx; 5 Departamento de Ciencia y Tecnología de Alimentos, División de Ciencia Animal, Universidad Autónoma Agraria Antonio Narro, Calzada Antonio Narro 1923, Buenavista, Saltillo, Coahuila C.P. 25315, Mexicoyajaira.lp@gmail.com (H.Y.L.-D.l.P.); maryhg12@yahoo.com (M.H.-G.); 6 Centro de Desarrollo de Productos Bióticos, Instituto Politécnico Nacional, Calle CeProBi Núm. 8, Colonia San Isidro, Yautepec, Morelos C.P. 62731, Mexico; rrendon@ipn.mx; 7 Facultad de Ciencias Agrotecnológicas, Universidad Autónoma de Chihuahua, Extensión Cuauhtémoc, Barrio de la presa s/n, Ciudad Cuauhtémoc, Chihuahua C.P. 31510, Mexico; aortega@uach.mx

**Keywords:** conservation, sausages, exoskeleton, TBARS

## Abstract

High molecular weight chitosan (≈322 kDa) was obtained from chitin isolated from *Brachystola magna* (Girard) to produced biodegradable films. Their physicochemical, mechanical and water vapor permeability (WVP) properties were compared against commercial chitosan films with different molecular weights. *Brachystola magna* chitosan films (CFBM) exhibited similar physicochemical and mechanical characteristics to those of commercial chitosans. The CFBM films presented lower WVP values (10.01 × 10^−11^ g/m s Pa) than commercial chitosans films (from 16.06 × 10^−11^ to 64.30 × 10^−11^ g/m s Pa). Frankfurt-type sausages were covered with chitosan films and stored in refrigerated conditions (4 °C). Their quality attributes (color, weight loss, pH, moisture, texture and lipid oxidation) were evaluated at 0, 5, 10, 15 and 20 days. Sausages covered with CFMB films presented the lowest weight loss (from 1.24% to 2.38%). A higher increase in hardness (from 22.32 N to 30.63 N) was observed in sausages covered with CFMB films. Compared with other films and the control (uncovered sausages), CFMB films delay pH reduction. Moreover, this film presents the lower lipid oxidation level (0.10 malonaldehyde mg/sample kg). Thus, chitosan of *B*. *magna* could be a good alternative as packaging material for meat products with high-fat content.

## 1. Introduction

Chitin is a linear biopolymer formed by units of N-acetylglucosamine [1]. It is the second most abundant biopolymer in nature after cellulose [2,3]. It constitutes the main structural exoskeleton of some species such as crustaceans and insects, it is also found in lower amounts in squid feathers and in other animals, besides plants, fungus and bacteria [4]. This biopolymer is of great importance since it is biocompatible, biodegradable and non-toxic for human beings [5,6]. Chitin presents some inconvenience due to its low solubility in most of the solvents, which reduces its applications and its structural and functional properties investigation [7]. Thus, it is necessary that chitin be deacetylated to obtain chitosan, which implies hydrolysis of the aceto-amide groups of chitin, the main functional group [4,8]. Then, chitosan is a co-polymer referred to a series of chitin derivates obtained after its partial deacetylation, not only at different degrees but at a different molecular weight [2]. At deacetylation degree upper to 50% (mol), the product turns soluble in acid conditions, due to amino group protonation in glucosamine C-2 position, which turns it into a more versatile biopolymer [9]. Chitosan presents a greater amount of functional and biological properties, with several potential applications related to its acetylation degree and molecular weight [10]. Because of that, chitosan has several technological applications since it can be used in the form of micro and nano-spheres, sponges, hydrogels, powder and films [1]. One of the most popular forms in which chitosan has been used is as coatings and biodegradable films, which reduces rapid moisture losses, prevents microbial growth and lower lipidic oxidation in food products, both fresh or processed, favoring food quality assurance and reducing environmental impact at the same time [11].

Chitosan films have been utilized on vegetables and fruits, such as tomatoes [12] and soursop [13], respectively. It has also been applied to fresh flesh and meat products [14,15,16,17]. Sausages are meat products that require refrigeration prior its sale and to be ready to cook, being really popular for its convenience and freshness [18]. Sausages are a complex food matrix, composed mainly of water, proteins, lipids, carbohydrates and species, so microbial and chemical alterations can occur while stored [19]. Its high lipidic content and packing in oxygen semi-permeable materials allows that the main spoilage is the lipidic oxidation not the microbial, which impact its nutritional and sensory properties [19,20]. In order to prevent this undesirable effect, chitosan has been used in these meat products. Sagoo et al. [21] reported a life shelf increment of 7 to 14 days in sausages submerged in a chitosan solution (0.05%, 0.25% and 0.50%) and in a saline solution (0.9%) at 6.2 pH. Alemán et al. [22] covered fish sausages (Alaskan pollock) with a mixture of fish jelly and commercial chitosan and up to 42 days stability (15 days more than sausages covered with commercial plastic). Arslan and Soyer [23] evaluated chitosan coatings on sausages fungal growth fermented in dry, concluding that chitosan (1%) coating reduced up to 2 logarithmic cycles yeast and fungus counts, delayed oxidation reactions and preserved sausage color and flavor. 

Chitosan films properties are influenced by several factors such as solvent (type and concentration), plasticizer and the presence of co-polymers and additives; nevertheless, chitin source and chitosan properties (molecular weight and deacetylation degree) also affect the physicochemical and microbiological properties of its derived films [24]. Most of the studies performed in meat products, using chitosan films, have employed commercial chitosans, which are obtained from maritime industry wastes [25]; since that, current research studies have been oriented to explore the properties of chitin and chitosan obtained from other sources available in nature, such as grasshoppers and other insects [25,26]. In this regard, Monter-Miranda et al. [27] characterized physicochemical, morphological and structurally, chitin and chitosan obtained from *Brachystola magna* (Girard), a polyphagous insect (grasshopper) which attacks corn and bean crops from the northern part of Mexico, causing large economic losses by reducing the production of these crops, and reported that the chitosan obtained from this insect presents chemical similarities with commercial ones. Recently this research group reported the feasibility of obtaining low molecular weight chitosan films from *Brachystola magna* and *Tenebrio molitor* insects and their physicochemical evaluations compared with different molecular weight commercial chitosan films [28]; despite that, so far, there are no assays on higher molecular weight chitosan biodegradable films obtained from *B. magna* insects and their evaluation on meat products inlay type (sausages). Thus, the objective of this research was to analyze the physicochemical, mechanical and water vapor permeability properties of films made from chitosan obtained from *B. magna* (Girard) and to evaluate the effect of these films on the quality attributes of sausages during refrigerated storage.

## 2. Results and Discussion

### 2.1. Molecular Weight of B. magna Chitosan

Chitosan molecular weight from *B. magna* (calculated by HPSEC) was 332.1 ± 5.75 kDa, and according to commercial specifications, could be considered as high molecular weight chitosan. This datum is higher than the value (3.22 kDa) reported by Kaya et al. [29] in chitosan obtained from scorpion, which was measured by capillary viscometer. Monter-Miranda et al. [27] reported the value of 25.8 ± 9.7 kDa measured by HPSEC for chitosan obtained from *B. magna* (Girard). Recently, Saenz-Mendoza et al. [28] had also reported similar values, despite that, these researchers established the molecular weight through capilar viscosimetry technique, obtaining an average viscosimetric molecular weight. Chitosan molecular weight differences found may be attributed to different factors such as obtention source, analysis or methods performed for its determination, and de-acetylation treatment conditions (type and reagents concentrations, time, temperature, alkaline steps repetitions, atmospheric pressure, particle size and chitin-solvent relation) [9,26].

### 2.2. Chitosan Films Characterization

#### 2.2.1. Color Evaluation

**Figure 1:**
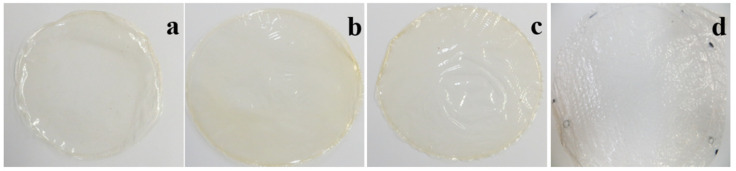
Photographs of chitosan films obtained from low molecular (**a**), medium molecular (**b**), low molecular weight (**c**) and *Brachystola magna* chitosan films (**d**).

The process before the production of the films, is known as filmogenic solution (FS) formation. In this sense, all FS formed homogenous films, flexibles, uniforms, porous less and fracture less, which were easily removed from the Petri dish (Figure 1).

In general, there were significant differences (*p* < 0.05) in some color variables between the films (Table 1). The L* value oscillated between 93.81 (CFMh) and 96.66 (CFMl); while CFBM got an intermediate value (95.15). These L* values are greater than those reported by Rotta et al. [30] in commercial chitosan films (L* = 48.39) at 2% (*w*/*w*), but similar to those reported by Souza et al. [31] in high molecular weight (L* = 91.8) commercial chitosan at 1.5% concentration (*w*/*v*). Regarding to variable b* (yellow coordinate), from 3.22 to 7.39, these are superior to those reported by Souza et al. [31] (b* = 1.6) and Rotta et al. [30] (b* = 1.8) in commercial chitosan films. On the other hand, values for variable a* (reddish coordinate) in these films (−0.05 to −0.93) were higher than those reported by Souza et al. [31] in high molecular weight chitosan (a = −1.7); despite, coordinate a* values in CFMl (a* = −0.80) and CFMh films (a* = −0.93) were lower than the ones reported by Rotta et al. [30] in chitosan films which, were also lower to those observed in CFMm (a* = −0.05) and CFBM (a* = −0.12) films. The total color difference (*ΔE) ranging between 5.98 and 1.31 with the following decreasing order: CFMh > CFBM > CFMl > CFMm. The highest values for *ΔE were observed in the CFMh (5.98) and CFBM (3.34) films, both obtained from high molecular weight chitosans, although of different origin. However, color difference was only significant (*p <* 0.05) for films obtained from commercial high molecular weight chitosan, and the *ΔE value obtained is considered as a perceptible color change. These color variations may be attributed to the concentration and type of components used, such as added plasticizer, chitosan molecular weight, deacetylation degree and films storage conditions [32,33,34]. In the practical sense, it can be indicated that CFMl, CFMm and CFMh film presented a light-yellow coloration and that the CFBM films were colorless (Figure 1). This is besides shown with the results for the chroma and °hue variables, where CFBM films exhibited a lower chroma value (5.12) in comparison with the other films and with a 90.5 °hue, indicating a lower °hue as well as lower color saturation. Finally, this can be considered an advantage since CFBM may be an appropriate packaging material that permits the observation of the coated product and, in such condition, the meat product (sausage).

#### 2.2.2. Water Vapor Permeability, Solubility, Moisture and Thickness

Water vapor permeability (WVP) is considered one of the main barrier properties in food packaging materials and should preferably be as low as possible. Since natural biopolymer films tend to be hydrophilic, the determination of WVP is essential to assess their feasibility and potential applications. [35]. Commercial chitosan films presented WVP values (Table 1) which increased from 16.06 to 64.30 × 10^−11^ g/m s Pa in a meaningful manner (*p* < 0.05) as molecular weight chitosan reduced. In general, the WVP of chitosan films decreases as the molecular weight of chitosan increases [28,36]. This behavior can be related with a greater amount of water vapor permeating the film. CBFM film outstands since it presented the lowest permeability value (10.01 × 10^−11^ g/m s Pa). It may be due to *B*. *magna* chitosan showed a high de-polymerization degree in chitosan chains because of thermal-alkaline treatment [37,38]. This promotes a larger number of glucans’ fractions, which may interact with the plasticizer (glycerol), forming matrices of greater homogeneity and superior compaction, which impacted in lower WVP. Regarding to nowadays researches on chitosan films WVP, Bof et al. [37] obtained films from commercial low, medium and high molecular weight chitosan dissolved in acetic acid. They reported WVP values (in the order of 1 × 10^−11^ g/m s Pa) of 41.4 ± 0.26 for low molecular weight chitosan, 33.8 ± 0.11, for medium molecular weight and 4.55 ± 0.6 for high molecular weight, these results are really close to the ones found in the present study.

Solubility values for chitosan films from *B*. *magna* (CFBM) (determined as percentage) were higher (49.11%) in comparison with commercial chitosan films (Table 1), which ranged from 14.48% to 23.30%. These values were more than twice and are related to the molecular weight, deacetylation degree and acetic acid (or any other organic acid) used for chitosan protonation within its dissolution stage before filmogenic solution formation [37]. In a study carried out by Kim et al. [39] in which high and low molecular weight commercial chitosans were evaluated dissolved in different organic acids, it was found that low molecular chitosan presented 29.5 ± 1.05% solubility, whereas for high molecular weight chitosan, this value was 27.6 ± 2.77%. These results agree with those obtained in this research since samples CFMl, CFMm and CFMh had 20.64%, 14.48% and 23.30% values, respectively. Solubility percentage results indicate that commercial chitosan films (independently of molecular weight) can be considered as biodegradable materials because of its water-soluble nature; even more, it can be considered that the result obtained from *B*. *magna* chitosan films (CFBM), which presented greater solubility values, may guaranty its biodegradability in high moisture environment [40]. The hydrophilic nature of polysaccharide-based films can limit their application for food packaging; however, they usually offer an excellent oxygen barrier to prevent the oxidation of lipids and other oxygen-sensitive components present in food [41]. Another advantage of highly soluble films is that they can be used in foods in which it is necessary to solubilize the coating before consuming the product [42].

Regarding the moisture content of the films, the values ranged between 32.10 and 47.30% (Table 1). The following behavior was observed in descending order (from highest to lowest): CFMh > CFMm > CFBM > CFMl; despite that, only commercial high molecular weight chitosan films (47.30%) presented significant differences (*p* < 0.05) when compared with the other formulations. In this last attribute, it is not clear the relation that the chitosan molecular weight can have regarding to films moisture, but it might be suggested that making a correlation of these values with their behavior of isothermal sorption would be the possibility of visualizing some objective relationship. Regarding to films thickness (Table 1), it was observed that in the commercial chitosan films as lower the molecular weight is, as smaller the thickness was, whereas in *B*. *magna* chitosan films (CFBM), an intermediate value was presented. In scientific literature there are not records about this behavior (as higher the chitosan molecular weight, as thicker the film is); on the other hand, it has been reported that the filmogenic solution viscosity and the amount of material poured (of filmogenic solution) in the Petri dish are factors that may directly influence the thickness of the film [37].

#### 2.2.3. Mechanical Properties Evaluation

The mechanical properties of chitosan films are shown in Table 2. The most important mechanical properties in packing material are tensile strength (TS, in MPa), elongation at break (%E) and elastic modulus (EM, in MPa). It was found in commercial chitosan films (CFMh, CFMm and CFMl) that as higher the chitosan molecular weight was, the TS increased significatively (*p* < 0.05), oscillating from 15.78 to 23.66 MPa. The %E values (25.70–39.55%) exhibited and inverse behavior, as higher as the chitosan molecular weight was as lower the %E. This behavior was similar to that previously reported in commercial chitosan films of different molecular weights [28,36].

It is worth mentioning that the mechanical properties of CFBM films (TS = 14.05 MPa, EM = 129.08 MPa and %E = 33.32%) were among within the values determined for commercial chitosan films (TS = 15.78 to 23.66 MPa, EM = 130.43 to 180.97 MPa and %E = 25.70% to 39.55%). Some researchers have reported the mechanical properties of chitosan films, similar to the ones reported in this research, for instance Butler et al. [43] evaluated low molecular weight chitosan films, in which values of TS ≈ 20 MPa and %E ≈ 42.3%, were obtained. In a recent study, Kerch and Korkhov [44] reported low molecular chitosan films values of TS ≈ 40 MPa; while %E, for low molecular weight films were 15%. Despite that, Vázquez-Briones and Guerrero-Beltrán [45] reported values of TS ≈ 5 MPa and %E = 29% for low molecular weight chitosan films, indicating that these values are related with chitosan concentration (%) employed for films elaboration.

On the other hand, the mechanical properties in the film CFBM were different from those found in the film CFMh, which may be inconsistent since both films were obtained from high molecular weight chitosan; however, the mechanical properties depend on elaboration conditions, chitosan source and its molecular weight, as well as the concentration of the components employed for the filmgenic solutions, and the glycerol or the plasticizers used [46]. Furthermore, in films obtained from chitosans isolated from insects, the mechanical properties can be affected by intramolecular interactions between chitosan chains and melanin [47].

### 2.3. Evaluation of the Effect of Chitosan Films on the Storage of Sausages

#### 2.3.1. Color Evaluation

Color parameters of covered sausages during storage are shown in Table 3. The color parameters, L* (brightness), a* (redness) and b* (yellowness) of the sausages in the initial time were 53.84, 17.00 and 12.50, respectively. Moreover, the chroma and hue angle value values were 21.05 and 36.12, respectively. The L* (≈53) variable indicated few variations on luminosity within treatments, despite that, bigger differences in the function of storage time. The L* variable (from 53.84 to 52.87) for sausages covered with *B*. *magna* chitosan films outstand since no significant differences were found (*p* ≥ 0.05) according to storage time. As previously mentioned, color variations on covered sausages with different chitosan films were minimum, and even more on sausages covered with CFBM. In general terms, significant diminutions were observed (*p* < 0.05) in a* (CFMl: from 17.00 to 14.95), b* (Ctrl: from 11.50 to 12.47 and CFBM: from 12.50 to 9.94), chroma (Ctrl: from 21.05 to 19.72, CFMl: from 21.05 to 19.13 and CFBM: from 21.05 to 18.70) and °hue (CFMl: from 36.12 to 38.63 and CFBM: from 36.12 to 32.08) variables when storage time was increased in sausages. These variations were attributed to biochemical changes that naturally occur on meat products, as recently reported by Zamudio-Flores et al. [48] in sausages covered with oxidized banana starch added with betalains. In general the color reduction in sausages during storage may be attributed to lipidic oxidation and malonaldehyde production, which causes sausage darkness [48,49].

On the other hand, the total color difference (*ΔE) is a parameter that gives us a more precise reference to the color change. According to the results, the *ΔE values increased (*p* ≤ 0.05) in all treatments during the storage time. The lower values were observed in control (from 2.66 to 1.54) and in the sausages covered with CFMh (from 1.80 from 2.27) and CFMl (from 1.93 to 2.30) films. For sausages covered with *B. magna* chitosan films, *ΔE values were from 2.96 to 2.96. Thus, although *ΔE values increased in all treatments, the color changes can be considered not perceptible (*ΔE < 5).

#### 2.3.2. Sausages Weight Loss, Moisture and pH

Weight loss (expressed as the weight percentage of the weight loss in function of time) and moisture content in uncovered and covered sausages with chitosan films is observed in Table 4. As it can be seen, all sausages, no matter the chitosan source used in the covering formulation, presented weight loss starting the 5 th evaluation day. This behavior prevailed until the end of the evaluation. These weight losses during storage time were higher in sausages covered with commercial chitosan films (CFMh: from 1.00% to 14.99%, CFMm: from 3.49% to 20.33% and CFMl: from 3.33% to 17.44%), even, greater than the control sausages (from 0.51% to 12.60%). These weight losses were greater in the sausages covered with CFMm and CFMl films, which can be attributed to their high WVP values (Table 1). This behavior prevailed until the end of the evaluation, indicating that these sausages suffered greater weight losses (CFMm = 20.33% and CFMl = 17.44%) on day 20. In this sense, sausages covered with the films CFBM maintained their weight without meaningful changes (*p* > 0.05) during the entire evaluation period, with a weight loss range from 1.24% to 2.38%. This behavior indicated that the water transference through the sausage to the exterior was minimal due to *B*. *magna* chitosan films presented WVP values significantly lower than CFMm and CFMl films (Table 1). This type of film is more effective than those based on other polysaccharides such as starch since prior studies performed by Zamudio-Flores et al. [48] reported higher weight losses (50%) in sausages covered with oxidized banana starch added with betalains. This is an interesting and promising field for further research studies related to *B*. *magna* chitosan films for food packaging.

On the other hand, in the moisture determinations (expressed as percentage) in the function of time (Table 4). The control did not present significant (*p* ≥ 0.05) variations in its moisture content during the storage time (from 71.02% to 68.88%). This behavior is attributed to moisture absorption from the environment since these products were cover less and storage in refrigeration. This moisture absorption was significant, or they maintained their initial moisture up to the end of the assay. On the other hand, slight reductions were observed (*p* < 0.05) in all the covered sausages compared to the control. During storage, the moisture content decreased significantly (*p* < 0.05) from 71.02% to 66.51%, from 71.02% to 67.01%, from 71.02% to 67.79%, and from 71.02% to 68.47% in the sausages covered with the CFMh, CFMm, CFMl, and CFBM films, respectively. In this field, several reports have been made. For example, Alemán et al. [22] applied films obtained from a mixture of chitosan (1%) with gelatin (1%) to fish sausages stored at 5 °C; the results showed that the sausages covered with the film registered lower moisture content during storage, which was attributed to water absorption for the films. In another study, do Amaral et al. [50] worked with sausages with different fats levels were covered with chitosan films (2% *w*/*w*) and moisture was determined at 0, 5, 10, and 15 days storage at 4 °C. As a result, moisture changes were low in the different samples. These percentages random between 61 and 66% (in sausages with the greatest fat content); these data are similar to those presented in the present assay.

The pH level obtained from sausages covered with commercial and *B*. *magna* chitosan are shown in Table 4. According to these results, the initial pH decreased with the storage time in all treatments (*p* < 0.05). During the storage, the pH values ranged from 6.21 to 6.04 for CFMh, from 6.21 to 6.06 for CFMm and from 6.21 to 6.28 for CFMl sausages. However, sausages covered with CFBM presented stable pH values (from 6.21 to 6.30) during all the study period (from 0 to 20 days of storage) at refrigeration conditions. On the other side, control sausages (uncovered), exhibited evident pH reductions (from 6.21 to 5.95) according to the storage period. Several researchers have attributed pH variations (in sausages) to microbial growth [48,51]. Some others have observed that pH lowering is associated with an increment in lactic acid production, specifically caused by the development and growth of bacteria from the generous *Lactobacillus*, regularly found in fresh flesh [48,52,53]. Another study, carried out by Lekjing [54], reported pH values obtained from sausages covered with chitosan at 2% (*w*/*v*) in a period from 0 to 25 days, varying from 6.27 to 6.12, respectively. These data are really close to the ones in this study, indicating that *B. magna* chitosan films can be used as an appropriate packaging to maintain sausages quality. 

#### 2.3.3. Texture Profile Analysis (TPA)

In general terms, TPA is an evaluation of great interest performed to food since the texture is one of the most important quality attributes used by consumers to judge or accept the products to be consumed [55,56]. In sausages, the texture may be affected unfavorably since factors like moisture, temperature (refrigeration conditions) and the material used for packaging (generally elaborated with synthetic materials) impact the food inner structural properties [56,57].

**Figure 2:**
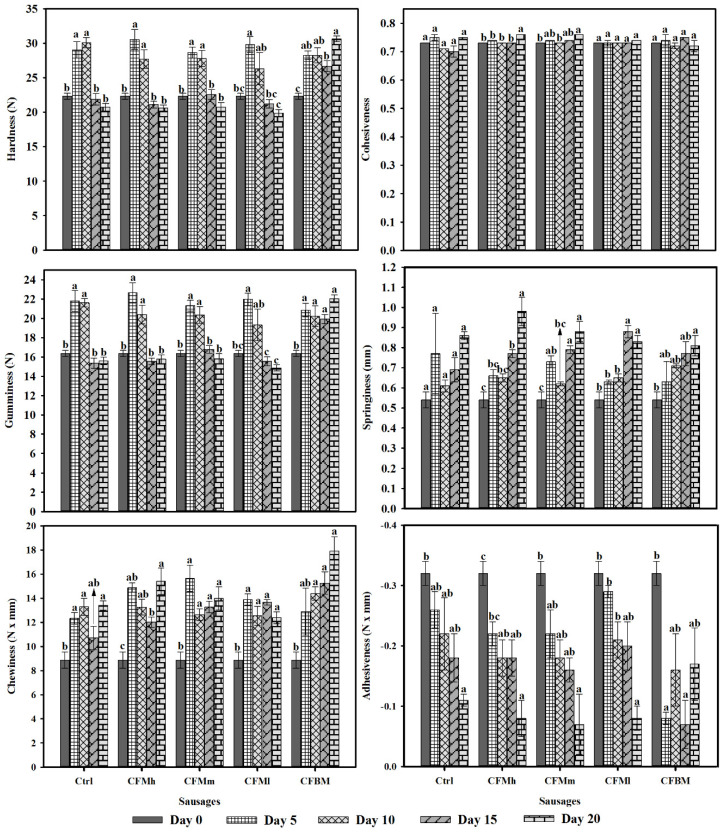
Texture profile analysis in covered sausages with chitosan *Brachystola magna* films (CFBM) and commercial high (CFMh), medium (CFMm) and low (CFMl) molecular weight chitosan films, as well as uncovered sample (Ctrl) during storage (0–20 days) at refrigeration (4 ± 1 °C). Results are arithmetic mean from at least three repetitions ± standard error. Bars connected by the same lowercase letters (a–c) in treatments are not significantly different among storage times (*p* ≥ 0.05) by Tukey´s test.

These evaluation results are shown in Figure 2. In general, chitosan films did not modify in significant manner (*p* > 0.05) sausage texture attributes, which could also be physically observed; despite that, significant variations (*p* ≤ 0.05) were determined in the same treatment in function to storage period. These variations were mainly evident on hardness attribute, in which considerable increments were observed at 5 and 10 days storage, and after that lower at the greater evaluation time; despite that, the determinations on day 20 did not reduce in a significant manner (*p* ≥ 0.05) from the data presented at the beginning of the essay (time 0). Cohesiveness showed a significant increase (*p* < 0.05) from 0.73 mm to 0.76 mm in CFMh and CFMm sausages during the storage time, respectively. Regarding elasticity, initial value for all treatments was 0.54 mm. Compared with the control, at the end of the assay (20 days) this parameter increased significantly (*p* < 0.05) in all the sausages covered with films (0.81 mm and 0.98 mm). This behavior can be related to the loss of moisture to the environment or the water absorption by the film [22]. The variation of the texture parameters between treatments and over time makes it difficult to conclude their behavior; however, one of the parameters most discussed in this type of study is hardness. In the sausages covered with *B*. *magna* chitosan (CFBM) the hardness increased significantly (*p* < 0.05) from 22.32 N to 30.63 N in function with the storage time. These last results agree with values reported by Zamudio-Flores et al. [48] according to the progressive increment of the hardness variable in function of storage time in sausages, and these increments would be attributed to natural biochemical process which takes place in meat aging more than moisture losses [48,58,59]. Some researchers have reported that coatings and films based on hydrocolloids or polysaccharides can cause variations in texture properties due to carbohydrates presence, as starch may hold back moisture in processed meats [48,58,59,60,61].

#### 2.3.4. Content of Thiobarbituric Acid Reactive Substances (TBARS)

Lipidic oxidation is one of the most important chemical reactions in food chemistry since poly-unsaturated fatty acids are highly susceptible to rust in the presence of molecular oxygen [50]. In this sense, meat products such as sausages are highly perishable; this type of reaction may occur due to the presence of fats coming from the raw material used to elaborate them. Table 5 presents values of the reactive species to thiobarbituric acid (TBARS) determined as monoaldehyde (MDA) mg per dry sample kg (mg MDA/kg).

At comparing values obtained from sausages covered with commercial chitosan vs. sausages covered with *B. magna* chitosan, similar results were found in films obtained from commercial low molecular weight chitosan. No significant differences were observed between all the treatments, but there were increases in TBARS within the same treatment as a function of time. Regarding the initial TBARS concentration (0.02 mg MDA/kg), all samples showed significant changes (*p* < 0.05) in their MDA concentration on the 5 th day of storage, ranged between 0.06 and 0.7 mg MDA/kg. At ten days of storage, a not significance (*p* ≥ 0.05) increase in TBARS was observed in control (from 0.06 to 0.09 mg MDA/kg), CFMh (from 0.07 to 0.09 mg MDA/kg) and CFMm (from 0.06 to 0.09 mg MDA/kg) sausages; this trend was maintained up to 15 days of storage. At the end of the assay, after 20 days of storage, the uncovered and covered sausages, regardless of the type of chitosan, showed a significant increase (*p* < 0.05) in the concentration of TBARS ranged from 0.10 to 0.12 mg MDA/kg. The highest values were registered in control (0.12 mg MDA/kg) and the CFMh (0.12 mg MDA/kg) sausages. Intermediate values were recorded in the CFMm (0.11 mg MDA/kg) and CFMl (0.11 mg MDA/kg) sausages, while the lowest concentration was observed in the sausages covered with the *B*. *magna* chitosan films (0.10 mg MDA/kg). There are several studies where chitosan coatings or films have been used to extend the shelf life of sausages; only a few of them are mentioned below. Soultos et al. [62] applied chitosan films, as coatings at a concentration of 0.5% and 1% (*w*/*w*) in pork sausages. Sausages were stored at 4 °C for 28 days. These researchers reported MDA contents ranging 0.1034 up to 0.5733 (mg MDA/g) from 0 to 21 evaluation days, respectively (for a sausage covered with a low concentration of chitosan) and from 0.1034 up to 0.4094 (mg MDA/g) for sausages covered with higher concentrations of chitosan. Siripatrawan and Noipha [63] reported results obtained from pork sausages covered with low molecular weight chitosan (2% *w*/*v*) films. A progressive increase in TBARS from 4 to 20 days of storage at 4 °C; however, from the 10 th day, the concentration was higher in sausages without films (≈0.13 mg MDA/kg) than covered (≈0.10 mg MDA/kg). In another study, Lekjing [54] applied a chitosan-based coating to increase the shelf life of cooked pork sausages in refrigerated storage (4 °C) for 25 days. The authors observed increases in TBARS concentrations since the 5 th day, which was sustained until the assay end. However, compared with de control (uncovered sausages), covered sausages presented less oxidation of lipids. In a more recent study (in non-refrigerated products), Arslan and Soyer [23] applied chitosan solutions (0.2–1% *w*/*v*) on the surfaces of cooked pork sausages aged 12 days (20–22 °C). After 12 days of storage, the TBARS concentration in the chitosan-coated sausages (55 ± 0.01 to 0.58 ± 0.01 mg MDA/kg) was lower than untreated sausages (0.80 ± 0.04 mg MDA/kg). According to the literature, a lower concentration of TBARS in sausages with chitosan coatings and films has been related to the antioxidant properties of chitosan and its low oxygen permeability [19,23,63]. The concentrations of TBARS in the previous studies differ from those found in Table 5. These differences may be due to the composition of the product, specifically the concentration and type of fat used in the formulation and the techniques and sensitivity of the equipment used in the test. However, these studies allowed us to outstand that *B. magna* chitosan films facilitate sausages conservation at delaying lipidic oxidation during storage in refrigeration conditions.

## 3. Materials and Methods

**Figure 3:**
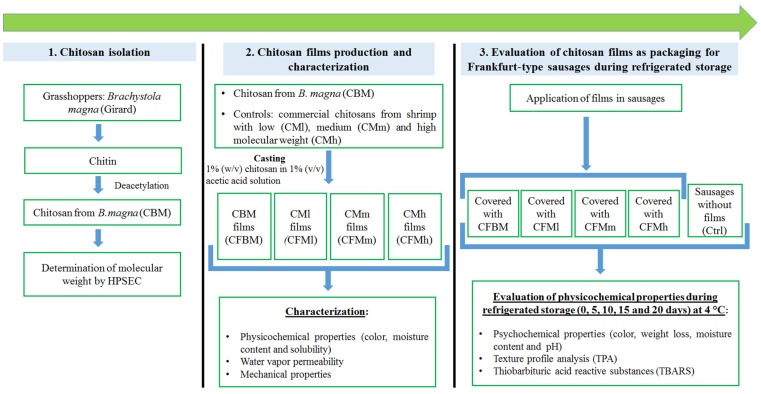
Description of the general methodoloy.

The general methodology applied in the present study is shown in Figure 3. Chitosan was isolated from *Brachystola magna* (Girard) grasshoppers and its molecular weight was measured. Biodegradable films were obtained, their physicochemical, mechanical and water barrier properties were compared against commercial chitosan films with different molecular weights (low, medium and high). Finally, Frankfurt-type sausages were covered with both *B*. *magna* chitosan films and commercial chitosan films; as a control, uncovered sausages were used. The effect of chitosan films on the shelf life of refrigerated sausages was evaluated by monitoring the quality attributes at different times (0–20 days).

### 3.1. Materials

*Brachystola magna* (Girard) insects were recollected at Valle de Allende, Chihuahua (Mexico) in bean crops and feeding lands. Once collected, they were washed at least two times with distilled water to remove contaminants and they were dry in an oven (Fine PCR, model combi-SV12DX, Daigger, Vernon Hills, IL, USA) at 60 ± 1 °C.

Reagents used for extraction were analytic degrees and were obtained from Sigma-Aldrich (Toluca, Mexico State, Mexico). As controls were used chitosans from shrimp with low molecular weight (named as CMl) (CAS 9012-76-4 product key 448869-250G), medium molecular weight (named as CMm) (CAS 9012-76-4, product key 448877-250G), and high molecular weight (named as CMh) (CAS 9012-76-4, product key 419419-250G) from Sigma-Aldrich (Toluca, México State, México).

Sausages type Frankfurt, BAFAR™, were purchased in a local market (Cuauhtémoc City, Chihuahua, Mexico). According to the specifications described by the manufacturer, sausages contained 44% turkey flesh, 43% water, 11% soybean oil, 1% sodium chloride, 0.4% sodium phosphate, 0.2% red pepper, 0.2% powder garlic, 0.02% ascorbic acid and 0.01% sodium nitrate. Nutritional values were ≈25% total carbohydrates, 22% protein and 4% fat. Sausages were purchased two days after being elaborated to have a fresh product. 

### 3.2. Chitin Isolation and Chitosan Obtention from Brachystola Magna Flour

#### 3.2.1. Chitin Isolation

Chitin was isolated through Liu et al. [55] method with some amends. For de-mineralization 10 g of powder (flour) were mixed with 500 mL of HCl 1 M, in 1 L Erlenmeyer flask, the mixture was heated 30 min at 100 °C. Later HCl was eliminated by filtration and the sample was washed with 500 mL of distilled water. Deproteinization was carried out by alkaline treatment with 500 mL of NaOH 1 M for 24 h at 80 °C in a 1 L Erlenmeyer flask. The product was washed three times with 500 mL of distilled water after HCl 0.1 M was added to neutralize pH. At last, chitin was dried in an oven model combi-SV12DX (Daigger Fine PCR, Vernon Hills, IL, USA) for 48 h at 30 ± 1 °C.

#### 3.2.2. Chitosan Isolation

Isolated chitin from *B. magna* was deacetylated to obtain chitosan (named as CBM) according to the methodology proposed by Zhang et al. [64], with some modifications. Chitin was solved with 300 mL of NaOH at 40%, adding 0.25 g of NaBH4 (as a reducing agent), with continuous stirring in a 1 L Erlenmeyer flask with a screw cap. The closed flask was heated at ≈108 °C for 2 h in a heating plaque (6795-220 PC 220 Corning Mexicana, S.A., Life Sciences, Monterrey, Mexico). It was then filtered (using a coffee pot filter) and washed twice with distilled water to remove debris trapped in the filter. The material detained was dissolved in a liquid solution of acetic acid at 2% with continuous stirring for 1 h, pH was adjusted at 9 in order to precipitate the chitosan, by using NaOH 2 M. Obtained chitosan was washed with distilled water to eliminate formed salts and finally lyophilized.

### 3.3. Molecular Weight Determination

Chitosan molecular weight was determined by high precision size exclusion chromatography (HPSEC) according to the methodology recently reported by Monter-Miranda et al. [27]. Dextrans standards (Sigma-Aldrich, St. Louis, MO, USA) of diverse molar mass (2.0 × 10^6^, 2.82 × 10^5^, 1.88 × 10^5^, 6.5 × 10^4^ and 4.0 × 10^4^ g/mol) were used to obtain a calibration curve. A sample of the biopolymer was solubilized in acetic acid at 0.25 M at a final concentration of 4 g/L. This solution was injected in a HPSEC equipped with a 500 ultra-hydrogel column (7.8 × 300 mm) and a light dispersion detector PL-ELS 1000 (Polymer Laboratories, Wilmington, DE, USA). The separation was done with 0.25 M acetic acid as mobile phase, at a 0.3 mL/min flow rate through an isocratic pump HP series 1100. The analysis was carried out by triplicate.

### 3.4. Chitosan Biodegradable Films Elaboration and Its Conditioning

Biodegradable films were produced according to the method reported by Srinivasa et al. [65] with some modifications. One percent (*w*/*v*) chitosan dispersion was prepared using glycerol as plasticizer. For this, chitosan was solved in an acetic acid solution (1%, *v*/*v*). After that, the sample was stirred up to having a homogenous filmogenic solution, it was then heated at 60 °C for 30 min, later it was cooled at room temperature (25 ± 5 °C) for 30 min with continuous stirring, then glycerol was added (at a concentration of 30% based on chitosan) and the mixture was stirred for 30 more min. Afterward, 50 g of the sample was poured into a 150 × 15 mm Petri dish. They were cooled at room temperature (25 ± 5 °C) in a dry place until the film could easily be detached from the dish. The films were conditioned on a 10 × 10 cm metal mesh in a desiccator containing a saturated solution of NaBr (50 ± 7 % HR; 25 °C) for at least 48 h.

### 3.5. Color, Moisture, Solubility and Mechanical Properties of Chitosan Films

Film color was evaluated in the CIE L*a*b* scale, using a colorimeter (Minolta CR-300, Minolta, Co., Ltd., Osaka, Japan) as reported by García-Tejeda et al. [66]. The total color difference (*ΔE) of the films was calculated by using the Equation (1)

(1) *ΔE=[(L1* − L2*)2+(a1* − a2*)2+(b1* − b2*)2]12

where L*_1_, a*_1_ and b*_1_ are the color parameter values of the standard white plate (L* = 96.13, a* = 0.04 and b* = 1.98) and L*_2_, a*_2_ and b*_2_ are the color parameter values of the sample. 

Moisture and solubility determination were performed in conditioned films, through the methodology reported by Zamudio-Flores et al. [67], and the solubility was estimated according to the method reported by Kim et al. [39] both analyses were reported in percentage (%). Films mechanical properties, such as tensile strength assessment (TS), elongation at break (%E) and elastic modulus (EM) were determined according to the method reported by Zamudio-Flores et al. [68], which is based in the ASTM standard method 882-95 [69].

### 3.6. Films Water Vapor Permeability

Water vapor permeability (WVP) was determined according to ASTM standard method E-9680 [70]. Films were cut in a circular shape and were set in aluminum cells (6.3 cm diameter and 1.5 cm depth) with 12 g of anhydrous silica gel as desiccant material (relative humidity, RH ≈ 0%). Cells were placed in a desiccator containing a NaCl saturated solution (RH ≈ 75%) at 25 ± 2 °C. Cells weight variation was graphed vs. time. Registered data adjusted to a linear regression model and the water transmission rate (WTR) was calculated from the slope (g/s), and the effective permeation area (0.0031 m^2^) (Equation (2)). The WVP (g/Pa h m) was determined according to Equation (3).

(2) $$\mathrm{WTR}=\left(\frac{\Delta w}{\Delta t}\right)\frac{1}{A}$$

(3) $$\mathrm{WVP}=\frac{\left(\mathrm{WTR}\right)\left(e\right)}{\Delta P}$$

where Δw is the weight change in the cell (g) in the function of time Δt (s), A is the exposed area of the film in the cell (m^2^), *e* is the film thickness (m) and ΔP is the gradient of water vapor partial pressure (Pa) in the desiccator and inside the cell (which was from 1730 Pa).

### 3.7. Evaluation of the Effect of Chitosan Films on the Storage of Sausages

The method reported by Zamudio-Flores et al. [48] was used to evaluate the effect of chitosan films on the quality characteristics of sausages, with modifications. Sausages covered with films (treatments CFMl, CFMm, CFMh, and CBM) and without films (control sample stated as Ctrl) were separated and randomly grouped in five lots of 2 kg. Sausages covered with the films were tied up at the ends with rubber bands in order to make sure that there were no air leaks or cracks. Sausages were stored in refrigeration at 4 ± 1 °C and RH ≈ 25% within 20 days. Evaluations were made at 0, 5, 10, 15 and 20 days of storage so as to evaluate physicochemical and texture changes. For the different tests, sausages of each lot were removed from the refrigerator and they were eliminated after the test. All tests were carried out in triplicate.

### 3.8. Color, Weight Loss, pH and Moisture Evaluation in Sausages during Storage

Storage sausages were evaluated in intervals of 5 days in the function of color, weight variation, pH, and moisture control. The color was measured in CIELab scale, with a Minolta colorimeter CR-300 (Minolta, Co., Ltd., Osaka, Japan). Equipment was calibrated with white standard and D65 was used with a 10° observation angle. Films were separated from the sausage and lectures were taken in three aleatory points on sausage surface. The total difference color (*ΔE) values were calculated according to the procedure previously described in Section 3.5 using Equation (1), where values L*_1_, a*_1_ and b*_1_ are the color parameter values of the fresh sausage at day 0, and L*_2_, a*_2_ and b*_2_ are the color parameter values of the sample at different times. Measurements were done in triplicate in each lot. The weight loss was carried out in a Scout Scale Pro SP401 (Ohaus, Co., Pinebrook, NJ, USA). Later, sausages were liquefied in an immersion mixer Taurus Robot 180 (Taurus, Oliana, Lleida, Spain), 5 g were taken and mixed with 45 mL of distilled water so as to determine pH with a potentiometer (HI 221 model, Hanna Instruments, Woonsocket, RI, USA Mauritania) [39]. Moisture content was determined following 952.08 AOAC official method. Five g of sausage were set in an oven (1350 GM, VWR Scientific Inc., Bristol, CT, USA) at 105 °C, up to have a weight loss of 0.0001 g. The moisture content of the samples (%) was calculated as the weight difference between humid and dry weight. It was used to correct the values of the affected variables due to sausage dehydration.

### 3.9. Sausage Texture Evaluation

Sausages texture changes (analyzed every 5 days) was evaluated with a texturometer (TAXT2 i-Plus, Stable Micro Systems, Surrey, UK) according to the method reported by Zamudio-Flores et al. [48]. Sausages were conditioned at 57% RH (with a saline solution NaBr) at 20 ± 2 °C for at least 60 min before the analysis. The texture profile analysis (TPA) was based on the double bite method. An aluminum cylindric probe (P0.25, θ = 0.25 inch) was used. Equipment (equipped with a 30 kg load cell) operation conditions were: Pre-test speed = 2 mm/s; after probe speed = 5 mm/s; load maximum = 2 kg, probe speed = 2 mm/s, distance = 8 mm, self-shot function was adjusted in 5 g. Measurements were performed using a force vs. time graph (as control variable), from which hardness (N) was established, springiness (mm), cohesiveness (dimensionless), gumminess (N), chewiness (N × mm) and the adhesiveness (N × mm) with exponent lite software™ (version 4.0).

### 3.10. Thiobarbituric Acid Level Measurement in Reactive Substances

Thiobarbituric acid reactive substances (TBARS) were quantified according to Botsoglou et al. [58] proposal. Sausages were liquefied in an immersion mixer Taurus Robot 180 (Taurus, Spain). Then, 1.5 g sample was mixed with 20 mL of water Millipore in an ultra-turrax (IKA, T18 Basic) at 500 rpm for 10 s. Five mL of trichloroacetic acid was added at 25% with slow and constant stirring (250 rpm, at 4 °C, for 15 min). The sample was centrifuged in a Beckman centrifuge Allegra 64-R (Indianapolis, IN, USA) at 13,500× *g* per 15 min at 4 °C. Supernatant (≈3.5 mL) was mixed with 1.5 mL of thiobarbituric acid aqueous solution at 0.6% (*w*/*v*) and was incubated at 70 °C for 30 min. The absorbance of the samples was measured at a λ = 532 nm in a Jenway spectrophotometer (model 6505, Jenway, Dunmow, Essex England). Quantitative data were obtained through the calibration curve elaborated with 1,1,3,3-tetraethoxypropane, as external pattern. Results expressed in malonaldehyde mg per dry sample g.

### 3.11. Statistical Analysis

Experiments were analyzed by a completely randomized statistical design. Samples analyzed in each experiment were determined at least in triplicate (*n* ≥ 3); otherwise, the sample size was indicated in each analysis performed. A one-way analysis of variance (ANOVA) was performed with the statistical program Sigma-Stat version 12.5 (Systat Software Inc., San Jose, CA, USA) and the means comparison with the Tukey test (*p* ≤ 0.05).

## 4. Conclusions

Chitosan films obtained from *Brachystola magna* (Girard) grasshoppers (CFBM) presented similar physicochemical and mechanical properties to those of commercial chitosans with different molecular weight. The CFBM films showed lower WVP values than commercial chitosan films, a desirable characteristic food packing oriented to contain foods with sensible oxygen components. Compared to commercial chitosan films, CFBM films limited weight loss in sausages. Commercial chitosan films exhibited higher weight loss values than the control (uncovered sausages); this could result from water absorption by the films. CFBN films delayed the drop in pH values, suggesting that probably these films limited the growth of acid-lactic bacteria. Moreover, compared with the rest of the treatments, sausages covered with CFBM recorded the lowest level of lipid oxidation at the assay end. On the other hand, although the total color difference in sausages covered with chitosan films, regardless of their origin, increased with time, no perceptive changes should be observed. This study suggests the feasible usage of high molecular weight chitosan obtained from *B. magna* (a specie considered a plague) to elaborate biodegradable films that may be used as packaging material for meat products. Besides, it would positively impact the rural sector at providing a new income source by giving added value to a resource only considered a prejudice both for commercial crops and subsistence.

## Figures and Tables

**Table 1:** Results of water vapor permeability (WVP), solubility, moisture, thickness and color, obtained from the evaluation of *Brachystola magna* and commercial chitosan films *.

Analysis	Film ^1^			
	CFMh	CFMm	CFMl	CFBM
WVP (× 10^−11^ g/m s Pa)	16.06 ± 5.10 ^b^	59.90 ± 11.2 ^a^	64.30 ± 1.00 ^a^	10.01 ± 2.20 ^b^
Solubility (%)	23.30 ± 0.90 ^b^	14.48 ± 1.51 ^b^	20.64 ± 3.84 ^b^	49.11 ± 1.43 ^a^
Moisture (%)	47.30 ± 0.56 ^a^	35.24 ± 1.34 ^b^	32.10 ± 1.50 ^b^	34.30 ± 0.66 ^b^
Thickness (µm)	52.83 ± 3.78 ^a^	50.37 ± 6.69 ^a^	36.26 ± 1.77 ^b^	43.21 ± 3.05 ^a^
Color	-	-	-	-
L*	93.81 ± 0.30 ^c^	96.54 ± 0.02 ^a^	96.66 ± 0.13 ^a^	95.15 ± 0.23 ^b^
a*	−0.93 ± 0.02 ^b^	−0.05 ± 0.01 ^a^	−0.80 ± 0.02 ^a,b^	−0.12 ± 0.14 ^a^
b*	7.39 ± 0.15 ^a^	3.22 ± 0.11 ^c^	3.69 ± 0.13 ^c^	5.12 ± 0.89 ^b^
Chroma	7.39 ± 0.21 ^b^	11.73 ± 1.54 ^a^	14.01 ± 1.12 ^a^	5.12 ± 0.90 ^b^
°hue	97.20 ± 0.14 ^a^	91.32 ± 0.24 ^b^	91.22 ± 0.35 ^b^	90.51 ± 1.18 ^b^
*ΔE	5.98 ± 0.02 ^a^	1.31 ± 0.11 ^b^	1.98 ± 0.14 ^b^	3.34 ± 0.77 ^b^

* Arithmetic mean from at least three repetitions ± standard error. Means connected by the same lowercase letters in the same row, for the same analysis are not significant different (*p ≥* 0.05). ^1^ Films abbreviations: CFMh = High molecular weight chitosan films, CFMm = Medium molecular weight chitosan films, CFMl = Low molecular weight chitosan films, CFBM = *Brachystola magna* chitosan films.

**Table 2:** Mechanical properties of *Brachystola magna* chitosan films compared to commercial chitosan films *.

Film ^1^	Mechanical Properties ^2^		
	TS (MPa)	EM (MPa)	%E
CFMh	23.66 ± 1.26 ^a^	180.97 ± 2.70 ^a^	25.70 ± 3.75 ^c^
CFMm	18.97 ± 2.41 ^b^	142.13 ± 4.91 ^b^	39.55 ± 3.62 ^a^
CFMl	15.78 ± 2.00 ^b,c^	130.43 ± 8.37 ^c^	38.03 ± 2.14 ^a^
CFBM	14.05 ± 0.79 ^c^	129.08 ± 1.78 ^c^	33.32 ± 1.80 ^b^

* Arithmetic mean from at least three repetitions ± standard error. Means connected by the same lowercase letters in the same column are not significant different (*p* ≥ 0.05). ^1^ Films abbreviations: CFMh = High molecular weight chitosan films, CFMm = Medium molecular weight chitosan films, CFMl = Low molecular weight chitosan films, CFBM = *Brachystola magna* chitosan films. ^2^ Mechanical properties are: TS = Tensile strength, EM = Elastic modulus, %E = Elongation at break.

**Table 3:** Color values of uncovered sausages (Ctrl) and those covered with chitosan films during storage (0–20 days) at refrigeration conditions (4 °C) *.

Color Variables	Day	Sausages ^1^				
		Ctrl	CFMh	CFMm	CFMl	CFBM
L*	0	53.84 ± 0.30 ^b^	53.84 ± 0.30 ^a,b^	53.84 ± 0.30 ^a^	53.84 ± 0.30 ^b^	53.84 ± 0.30 ^a^
	5	56.15 ± 0.17 ^a,A^	55.61 ± 0.18 ^a,A^	55.23 ± 0.27 ^a,A^	55.67 ± 0.29 ^a,A^	53.69 ± 0.33 ^a,B^
	10	54.56 ± 0.14 ^b,A^	53.63 ± 0.13 ^b,B,C^	54.28 ± 0.16 ^a,A,B^	53.38 ± 0.31 ^b,C^	53.36 ± 0.21 ^a,B,C^
	15	53.90 ± 0.22 ^b,A^	53.29 ± 0.31 ^b,c,A,B^	52.38 ± 0.23 ^a,b,B^	53.22 ± 0.17 ^b,A,B^	53.36 ± 0.41 ^a,A,B^
	20	53.44 ± 0.53 ^b,A^	51.41 ± 0.88 ^c,A,B^	49.40 ± 1.61 ^b,B^	52.98 ± 0.43 ^b,A,B^	52.87 ± 0.25 ^a,A,B^
a*	0	17.00 ± 0.38 ^b^	17.00 ± 0.38 ^a^	17.00 ± 0.38 ^a^	17.00 ± 0.38 ^a^	17.00 ± 0.38 ^a^
	5	18.12 ± 0.32 ^a,B^	17.13 ± 0.26 ^a,B,C^	17.23 ± 0.27 ^a,B,C^	16.78 ± 0.20 ^a,C^	16.12 ± 0.21 ^a,A^
	10	16.76 ± 0.13 ^b,A^	16.77 ± 0.29 ^a,A^	15.97 ± 0.23 ^a,A^	16.13 ± 0.37 ^a,b,A^	16.23 ± 0.24 ^a,A^
	15	16.11 ± 0.16 ^b,A,B^	16.18 ± 0.22 ^a,A,B^	16.54 ± 0.36 ^a,A^	15.38 ± 0.15 ^b,B^	15.75 ± 0.12 ^a,A,B^
	20	16.03 ± 0.14 ^b,A,B^	16.13 ± 0.29 ^a,A,B^	16.75 ± 0.64 ^a,A^	14.95 ± 0.38 ^b,B^	15.84 ± 0.34 ^a,A,B^
b*	0	12.50 ± 0.35 ^a,b^	12.50 ± 0.35 ^a^	12.50 ± 0.35 ^a^	12.50 ± 0.35 ^a^	12.50 ± 0.35 ^a^
	5	13.14 ± 0.37 ^a,A^	12.44 ± 0.15 ^a,A^	12.46 ± 0.58 ^a,A^	12.04 ± 0.16 ^a,A^	9.70 ± 0.18 ^b,B^
	10	12.16 ± 0.13 ^a,b,A^	12.59 ± 0.33 ^a,A^	11.58 ± 0.27 ^a,A^	12.36 ± 0.38 ^a,A^	9.78 ± 0.27 ^b,B^
	15	11.82 ± 0.18 ^b,A^	12.23 ± 0.55 ^a,A^	12.59 ± 0.34 ^a,A^	12.02 ± 0.15 ^a,A^	10.04 ± 0.20 ^b,B^
	20	11.47 ± 0.19 ^b,A^	11.50 ± 0.23 ^a,A^	12.27 ± 0.36 ^a,A^	11.95 ± 0.14 ^a,A^	9.94 ± 0.23 ^b,B^
Chroma	0	21.05 ± 0.52 ^ab^	21.05 ± 0.52 ^a^	21.05 ± 0.52 ^a^	21.05 ± 0.52 ^a^	21.05 ± 0.52 ^a^
	5	22.38 ± 0.47 ^a,A^	21.17 ± 0.28 ^a,A,B^	21.27 ± 0.51 ^a,A,B^	20.65 ± 0.24 ^a,b,B,C^	18.81 ± 0.26 ^b,C^
	10	20.72 ± 0.15 ^b,A^	20.97 ± 0.37 ^a,A^	19.72 ± 0.30 ^a,A,B^	20.32 ± 0.51 ^a,b,A,B^	18.95 ± 0.34 ^b,B^
	15	19.98 ± 0.24 ^b,AB^	20.29 ± 0.50 ^a,A,B^	20.78 ± 0.48 ^a,A^	19.51 ± 0.20 ^a,b,A,B^	18.67 ± 0.21 ^b,B^
	20	19.72 ± 0.12 ^b,AB^	19.81 ± 0.20 ^a,A,B^	20.77 ± 0.68 ^a,A^	19.13 ± 0.36 ^b,A,B^	18.70 ± 0.36 ^b,B^
°hue	0	36.12 ± 0.23 ^a^	36.12 ± 0.23 ^a^	36.12 ± 0.23 ^a^	36.12 ± 0.23 ^b,c^	36.12 ± 0.23 ^a^
	5	35.89 ± 0.32 ^a,A^	35.92 ± 0.30 ^a,A^	35.73 ± 0.99 ^a,A^	35.57 ± 0.23 ^c,A^	30.99 ± 0.31 ^b,B^
	10	35.86 ± 0.34 ^a,A^	36.82 ± 0.63 ^a,A^	35.86 ± 0.53 ^a,A^	37.37 ± 0.42 ^a,b,A^	31.04 ± 0.33 ^b,B^
	15	36.15 ± 0.18 ^a,A^	36.93 ± 0.86 ^a,A^	37.18 ± 0.30 ^a,A^	37.95 ± 0.19 ^a,C^	32.47 ± 0.31 ^b,B^
	20	35.49 ± 0.58 ^a,B^	35.42 ± 0.90 ^a,C^	36.19 ± 0.74 ^a,A,B^	38.63 ± 0.57 ^a,A^	32.08 ± 0.60 ^b,C^
*ΔE	0	0	0	0	0	0
	5	2.66 ± 0.37 ^a,A^	1.80 ± 0.19 ^a,A^	1.55 ± 0.27 ^a,A^	1.93 ± 0.21 ^a,A^	2.96 ± 0.25 ^a,A^
	10	0.86 ± 0.03 ^b,B^	0.55 ± 0.12 ^a,B^	1.47 ± 0.28 ^a,A,B^	1.06 ± 0.49 ^a,B^	2.88 ± 0.35 ^a,A^
	15	1.15 ± 0.22 ^a,b,A^	1.14 ± 0.44 ^a,A^	1.60 ± 0.29 ^a,A^	1.81 ± 0.23 ^a,A^	2.82 ± 0.30 ^a,A^
	20	1.54 ± 0.35 ^a,b,A^	2.27 ± 0.95 ^a,A^	4.50 ± 1.64 ^a,A^	2.30 ± 0.53 ^a,A^	2.98 ± 0.41 ^a,A^

* Arithmetic mean from at least three repetitions ± standard error. Means connected by the same lowercase letters in the same column for each treatment are not significantly different (*p* ≥ 0.05). Means connected by the same uppercase letters are not significantly different (*p* ≥ 0.05) between different treatments at the same day of evaluation. ^1^ Ctrl = Control sausage (not covered), CFMh = Sausage covered with high molecular weight chitosan films, CFMm = Sausage covered with medium molecular weight chitosan films, CFMm = Sausage covered with low molecular weight chitosan films, CFBM = Sausage covered with *Brachystola magna* chitosan films.

**Table 4:** Weight loss, moisture and pH values in sausages of uncovered sausages (Ctrl) and those covered with chitosan films during storage (0–20 days) at refrigeration conditions (4 ± 1 °C) *.

Analysis	Day	Sausage ^1^				
		Ctrl	CFMh	CFMm	CFMl	CFBM
Weight loss (%)	0	0	0	0	0	0
	5	0.51 ± 0.03 ^b,B^	1.00 ± 0.5 ^b,B^	3.49 ± 0.07 ^a,A^	3.33 ± 0.09 ^c,A^	1.24 ± 0.15 ^a,B^
	10	1.41 ± 0.37 ^b,C^	3.24 ± 0.91 ^b,C^	6.09 ± 0.70 ^a,B^	8.87 ± 0.40 ^b,c,A^	1.37 ± 0.32 ^a,C^
	15	8.76 ± 3.14 ^a,b,A,B^	14.15 ± 0.77 ^a,A^	16.25 ± 1.28 ^b,A^	10.82 ± 1.95 ^b,A,B^	1.99 ± 0.22 ^a,B^
	20	12.60 ± 2.64 ^a,A,B^	14.99 ± 0.90 ^a,A^	20.33 ± 3.46 ^b,A^	17.44 ± 2.39 ^a,A^	2.38 ± 0.30 ^a,B^
Moisture (%)	0	71.02 ± 0.07 ^a^	71.02 ± 0.07 ^a^	71.02 ± 0.07 ^a^	71.02 ± 0.07 ^a^	71.02 ± 0.07 ^a^
	5	70.88 ± 0.10 ^a,A^	69.99 ± 0.03 ^b,A^	70.35 ± 0.03 ^a,b,A^	70.33 ± 0.04 ^b,A^	69.72 ± 1.08 ^a,b,A^
	10	70.87 ± 0.07 ^a,A^	69.94 ± 0.10 ^b,B^	70.15 ± 0.05 ^a,b,B^	70.05 ± 0.11 ^b,c,B^	69.90 ± 0.30 ^a,b,B^
	15	69.40 ± 0.07 ^a,A^	68.76 ± 0.04 ^c,A^	68.26 ± 2.23 ^a,b,A^	69.82 ± 0.10 ^c,A^	68.51 ± 0.11 ^b,A^
	20	68.88 ± 2.01 ^a,A^	66.51 ± 0.19 ^d,A^	67.01 ± 0.13 ^b,A^	67.79 ± 0.18 ^d,A^	68.47 ± 0.11 ^b,A^
pH	0	6.21 ± 0.02 ^bc^	6.21 ± 0.02 ^c^	6.21 ± 0.02 ^c^	6.21 ± 0.02 ^c^	6.21 ± 0.02 ^c^
	5	6.30 ± 0.05 ^b,A^	6.49 ± 0.06 ^b,A^	6.46 ± 0.06 ^b,A^	6.32 ± 0.03 ^b,A^	6.45 ± 0.02 ^a,A^
	10	6.70 ± 0.01 ^a,C^	6.90 ± 0.00 ^a,A^	6.90 ± 0.01 ^a,A^	6.80 ± 0.01 ^a,B^	6.32 ± 0.02 ^b,D^
	15	5.72 ± 0.13 ^c,d,B^	6.27 ± 0.03 ^c,A^	6.19 ± 0.02 ^c,d,A^	6.19 ± 0.04 ^c,A^	6.26 ± 0.04 ^b,c,A^
	20	5.95 ± 0.03 ^d,C^	6.04 ± 0.02 ^d,B,C^	6.06 ± 0.02 ^d,B^	6.28 ± 0.03 ^b,c,A^	6.30 ± 0.02 ^b,c,A^

* Arithmetic mean from at least three repetitions ± standard error. Means connected by the same lowercase letters in the same column for each treatment are not significantly different (*p* ≥ 0.05). Means connected by the same uppercase letters are not significantly different (*p* ≥ 0.05) between different treatments at the same day of evaluation. ^1^ Ctrl = Control sausage (not covered), CFMh = Sausage covered with high molecular weight chitosan films, CFMm = Sausage covered with medium molecular weight chitosan films, CFMm = Sausage covered with low molecular weight chitosan films, CFBM = Sausage covered with *Brachystola magna* chitosan films.

**Table 5:** Quantification of TBARS (mg MDA/kg sample) in uncovered sausages (Ctrl) and those covered with chitosan films during storage (0–20 days) at refrigeration conditions (4 ± 1 °C) *.

Time (days)	Sample				
	Ctrl	CFMh	CFMm	CFMl	CFBM
0	0.02 ± 0.01 ^b,A^	0.02 ± 0.01 ^c,A^	0.02 ± 0.01 ^c,A^	0.02 ± 0.01 ^b,A^	0.02 ± 0.01 ^c,A^
5	0.06 ± 0.01 ^a,b,A^	0.07 ± 0.00 ^b,A^	0.06 ± 0.00 ^b,c,A^	0.07 ± 0.01 ^a,A^	0.06 ± 0.00 ^b,A^
10	0.09 ± 0.03 ^a,b,A^	0.09 ± 0.02 ^a,b,A^	0.09 ± 0.01 ^a,b,A^	0.07 ± 0.00 ^a,b,A^	0.06 ± 0.01 ^b,A^
15	0.10 ± 0.01 ^a,b,A^	0.09 ± 0.00 ^a,b,A^	0.10 ± 0.01 ^a,b,A^	0.07 ± 0.01 ^a,b,A^	0.09 ± 0.01 ^a,b,A^
20	0.12 ± 0.01 ^a,A^	0.12 ± 0.01 ^a,A^	0.11 ± 0.01 ^a,A^	0.11 ± 0.01 ^a,A^	0.10 ± 0.01 ^a,A^

* Arithmetic mean from at least three repetitions ± standard error. Means connected by the same lowercase letters are not significantly different (*p* ≥ 0.05) and indicate difference in the same treatment at different evaluation times. Means connected by the same uppercase letters are not significantly different (*p* ≥ 0.05) and indicate differences between treatments at the same evaluation periods. Ctrl = Control sausage (not covered), CFMh = Sausage covered with high molecular weight chitosan films, CFMm = Sausage covered with medium molecular weight chitosan films, CFMm = Sausage covered with low molecular weight chitosan films, CFBM = Sausage covered with *Brachystola magna* chitosan films.

## Data Availability

Data is contained within the article.

## Sample Availability

Samples of the compounds are not available from the authors.
